# Candidate genes for chromosomes 6 and 10 quantitative trait loci for age-related retinal degeneration in mice

**Published:** 2010-06-05

**Authors:** Diego G. Ogando, Kam D. Dahlquist, Mitra Alizadeh, Kannan Kunchithapautham, Jun Li, Nicole Yu, Matthew M. LaVail, Bärbel Rohrer, Douglas Vollrath, Michael Danciger

**Affiliations:** 1 Department of Biology, Loyola Marymount University, Los Angeles, CA; 2 Department of Ophthalmology and Neurosciences, Division of Research; Medical University of South Carolina, Charleston, SC; 3 Department of Genetics, Stanford University School of Medicine, Stanford, CA; 4 Beckman Vision Center, UCSF School of Medicine, San Francisco, CA

## Abstract

**Purpose:**

In a previous study, several quantitative trait loci (QTL) that influence age-related degeneration (ageRD) were identified in a cross between the albino strains B6(Cg)-Tyr(c-2J)/J (B6a) and BALB/cByJ (C). The Chromosome (Chr) 6 and Chr 10 QTL were the strongest and most highly significant loci and both involved B6a protective alleles. The QTL were responsible for 21% and 9% of the variance in phenotypes, respectively. We focused on these two QTL to identify candidate genes.

**Methods:**

DNA microarrays were used for the two mouse strains at four and eight months of age to identify genes that are differentially regulated and map to either QTL. Gene Ontology (GO) analysis of the differentially expressed genes was performed to identify possible processes and pathways associated with ageRD. To identify additional candidates, database analyses (Positional Medline or PosMed) were used. Based on differential expression, PosMed, and the presence of reported polymorphisms, five genes per QTL were selected for further study by sequencing analysis and qRT–PCR. Tumor necrosis factor, alpha- induced protein 3 (*Tnfaip3;* on a C57BL/6J (B6) background) was phenotypically tested. Single nucleotide polymorphisms (SNPs) flanking this gene were correlated with outer nuclear layer thickness (ONL), and eight-month-old *Tnfaip3*^+/−^ mice were tested for ageRD.

**Results:**

Polymorphisms were found in the coding regions of eight genes. Changes in gene expression were identified by qRT–PCR for Hexokinase 2 (*Hk2)* and Docking protein 1 (*Dok1)* at four months and for *Dok1* and *Tnfaip3* at eight months. *Tnfaip3* was selected for phenotypic testing due to differential expression and the presence of two nonsynonymous mutations. However, when ONL thickness was compared in eight-month-old congenic *Tnfaip3*^+/−^ and *Tnfaip3*^+/+^ mice, no differences were found, suggesting that *Tnfaip3* is not the quantitative trait gene (QTG) for the Chr 10 QTL. The GO analysis revealed that GO terms associated with stress and cell remodeling are overrepresented in the ageRD-sensitive C strain compared with the B6a strain with age (eight months). In the ageRD-resistant B6a strain, compared with the C strain, GO terms associated with antioxidant response and the regulation of blood vessel size are overrepresented with age.

**Conclusions:**

The analyses of differentially expressed genes and the PosMed database yielded candidate genes for the Chr 6 and Chr 10 QTL. HtrA serine peptidase 2 (*Htra2)*, *Dok1*, and *Tnfaip3* were deemed most promising because of their known roles in apoptosis and our finding of nonsynonymous substitutions between B6a and C strains. While *Tnfaip3* was excluded as the QTG for the Chr 10 QTL, *Dok1* and *Htra2* remain good candidates for the Chr 6 QTL. Finally, the GO term analysis further supports the general hypothesis that oxidative stress is involved in ageRD.

## Introduction

Age-related macular degeneration (AMD) is the most common cause of irreversible vision loss in the Western world [1]. It is a complex disorder where multiple genes and environmental factors determine the phenotype. The first factor that influences AMD is age. Age-related macular degeneration occurs predominantly in older individuals, typically those over 50 years of age. The most prevalent environmental risk factor is smoking [2]. Smoking, which is thought to cause oxidative stress, increases the risk of AMD, especially in individuals who carry a polymorphism in complement factor H^3^. Human genetics studies have identified multiple genes associated with AMD, including complement factors H *(CFH)*, complement factor B *(CFB),* and complement component 2 (*C2)*, Chemokine (C-X3-C motif) receptor 1 (*CX3CR1)*, Age-related macular susceptibility 2 (*ARMS2)*, HtrA serine peptidase 1 (*HTRA1)*, Apolipoprotein E (*ApoE)*, Vascular endothelial growth factor A (*VEGF-A),* and ATP-binding cassette subfamily A (ABC1) member 4 (*ABCA4*; for reviews, see [3,4]). The use of mouse models has confirmed the involvement of some of these genes in age-related retinal degeneration (ageRD) [3,4].

We have found that gradual, spontaneous ageRD occurs in many inbred strains of mice and often differs in rate among strains due to their genetic background [5,6]. In particular, BALB/cByJ (C) albino mice experience significantly more retinal degeneration as they age than B6(Cg)-Tyr(c-2J)/J (B6a) albinos [5]. This differential sensitivity is explained by genes present in quantitative trait loci (QTL) on Chromosomes (Chrs) 6, 10, 16, 14, 18, 12, 13, and 8 (listed in order of strength of influence on the phenotype) [5].

The goal of this study was to identify candidate genes that might explain this differential sensitivity to ageRD. We focused on the QTL on Chrs 6 and 10, as they are highly significant and involve B6a protective alleles (or conversely, C degenerative alleles) that are responsible for 21% and 9% of the phenotypic variance, respectively. This percent variance is large enough to allow for phenotype testing. We were guided by our working hypothesis that genes that are differentially regulated between strains (in particular at eight months of age) and are localized to the QTL are good candidate genes to explain the differential sensitivity, along with genes that have nonsynonymous coding region polymorphic changes between the strains. We performed a competitive microarray for the strains, database analysis (Positional Medline), reported SNP analysis, sequencing analysis, and qRT–PCR to identify and study candidate genes. Using knockout mice, one candidate gene was phenotypically tested as a candidate for the quantitative trait gene (QTG) of the Chr 10 QTL.

The Gene Ontology analysis of the microarray identified processes and pathways that may modulate ageRD.

## Methods

### Mice

Two albino mouse strains were used for this study: BALB/cByJ (C) and B6(Cg)-Tyr(c-2J)/J (B6a). Both were originally purchased from Jackson Laboratories (Bar Harbor, ME) and were maintained in our vivarium through many generations. The B6a mice were derived from C57BL/6J mice and are homozygous for a base pair substitution that inactivates the tyrosinase gene (c) making them isogenic with B6 and rendering them albino.

Tumor necrosis factor, alpha- induced protein 3 *(Tnfaip3)^+/−^* animals were generously provided by Dr. Averil Ma (University of California, San Francisco, CA). To allow for comparison with B6 mice, *Tnfaip3^+/−^* mice were backcrossed onto the B6 strain to N8-N10. As *Tnfaip3^−/−^* is lethal, N8-N10 congenic *Tnfaip3*^+/−^ and *Tnfaip3*^+/+^ mice were aged to eight months and sacrificed using CO_2_. Eyes were enucleated and used for outer nuclear layer (ONL) thickness determination. All mice were kept under a 12 h light-dark cycle with average light intensity in the cage of 2–7 ft-c. The temperature of the vivarium was maintained between 18 °C and 20 °C.

The following measures were applied to ensure equal light exposure for the animals: cages were kept on the lower shelves of freestanding racks and the cages were rotated by shelf and position on the shelf each week. Mice were maintained on a low-fat maintenance diet (15001 Rodent Laboratory Chow; Newco Distributors, Rancho Cucamonga, CA) with access to chow and water ad libitum. Mating pairs, pregnant mice, and mothers with pups had a higher fat diet (15015 Rodent Laboratory Chow; Newco Distributors).

All procedures were performed in accordance with the Association for Research in Vision and Ophthalmology (ARVO) Statement for the Use of Animals in Ophthalmic and Vision Research and were approved by the Loyola Marymount University Committee on Animal Research.

### Measurement of outer nuclear layer thickness

After the mice were aged to eight months, eyes were enucleated, fixed in a mixture of 2% formaldehyde and 2.5% glutaraldehyde in a phosphate buffer, embedded in an Epon-Araldite mixture, and bisected along the vertical meridian through the optic nerve head. To enable determination of the superior and inferior retina, the superior sclera was marked with a fine point marker before enuclation. A single 1 µm section was taken from the cut surface of one of the eyecup halves from each mouse and stained with toluidine blue, as previously described [7]. In each of the superior and inferior hemispheres, the ONL thickness was measured in nine sets of 108 three measurements each (a total of 27 measurements in each hemisphere). Each set was centered on adjacent 250 µm lengths of retina, with the first set centered 250 µm from the optic nerve head and subsequent sets located more peripherally. Within each 250 µm length, the three measurements were made at defined points separated from one another by 50 µm using an eyepiece micrometer. Since the retinal length in each hemisphere was 2.25–2.75 mm, the 54 measurements in the two hemispheres sampled regions of almost the entire retinal section [7,8].

### Single nucleotide polymorphism correlation with outer nuclear layer thickness

As mentioned above, there is a highly significant QTL on Chr 10 that explains the differential sensitivity to ageRD in both strains of mice [5]. The Chr 10 QTL maps the position 15.4–20.8 Mb. Polymorphisms in that region are highly associated with ONL thickness. We compared the ONL thickness of F2 animals from the original study [5] that were polymorphic for two single nucleotide polymorphisms (SNPs) that span *Tnfaip3*. The SNP rs4135995 is localized 1.4 Mb downstream of *Tnfaip3* and rs1348525 is localized 0.3 Mb upstream of Tnfaip3. A one-way ANOVA test, followed by pairwise multiple comparisons using the Holm-Sidak method, was applied to compare the ONL thickness of mice being homozygous B6a, heterozygous, or homozygous C for the mentioned SNPs.

### Microarray experiments

#### RNA preparation

Posterior eyecups from both C and B6a strains of mice (females) sacrificed at four and eight months of age were dissected, quick frozen in a dry ice/95% ethanol bath, and stored at −80 °C. Total RNA was isolated following homogenization of eyecups using TRIzol® solution (Invitrogen, Carlsbad, CA). To obtain a sufficient amount of high quality total RNA and to reduce biologic variability [9], eyecups from three animals (six eyes) were pooled for each sample. For each strain (C and B6a) and each age (four and eight months), four RNA samples were obtained. A total of 48 mice were used for the microarray experiment (12 animals for each genotype and time point).

#### Sample labeling and microarray hybridization

RNA was linearly amplified using a method similar to that described by Van Gelder, Eberwine, and coworkers [10-12]. Amplified RNA served as a template for the generation of cDNA labeled with fluorescent dye, Cy3, or Cy5. The four-month C samples labeled with Cy5 were hybridized with the four-month B6a samples labeled with Cy3 (four biologic replicates); the eight-month C samples labeled with Cy5 were hybridized with the eight-month B6a samples labeled with Cy3, for eight arrays (four biologic replicates). Another eight hybridizations were performed on a second set of arrays with the same RNA samples and the dye orientation reversed, yielding a total of 16 arrays in the experiment.

The mouse cDNA microarrays used here were manufactured at the Stanford Functional Genomics Facility (Stanford, CA) and contained 42,000 cDNA clones on glass slides representing ~25,000 unique genes. After hybridization, the arrays were washed according to standard procedures, scanned on an Axon 4000B scanner, and gridded using GenePix® Pro 5.0 software. Raw data were uploaded to the Stanford Microarray Database (SMD). Raw data and normalized data were submitted to the Gene Expression Omnibus (GEO). The GEO accession number is GSE18151.

#### Data processing and normalization

The raw intensity data for the two fluorescent channels (ch1 and ch2) were downloaded from the SMD and the log_2_ (ch2/ch1) ratios for all cDNA probes were calculated for all 16 chips. Lowess normalization was performed so that the intensity dependence of the log_2_ (ch2/ch1) ratio was corrected (with f=0.33) [13]. The parameter f is the Lowess fit applied to the log_2_(ch2/ch1) versus log_2_ (Sqrt [ch1xch2]) plot. Subsequently, log_2_ ratios in all chips were linearly re-scaled so that the standard deviation of log_2_ ratios, calculated as the standard deviation among all genes within each chip, are the same across chips and equal to the median value of standard deviation across the 16 chips. The dye effect (D) for each gene was estimated as follows: the average of normalized log_2_ ratios for the eight C-Cy5/B6a-Cy3 chips, plus the average of normalized log_2_ ratios of the eight B6a-Cy5/C-Cy3 chips is twice the Cy5/Cy3 effect (2D). The dye effect was removed and the B6a/C chips were flipped to C/B6a using the following procedure: subtracting D from the normalized log_2_ ratios for the eight C-Cy5/B6a-Cy3 chips and adding D to the negative of the normalized log_2_ ratios for the eight B6a-Cy5/C-Cy3 chips. Genes missing four or more values among the eight C-Cy5/B6a-Cy3 chips or among the eight B6a-Cy5/C-Cy3 chips were excluded. A total of 5,212 genes were removed from analysis.

The following comparisons were performed: (1) the C/B6a difference in all 16 chips to determine whether the average of the 16 adjusted C/B6a values is significantly non-zero, using a one-group *t*-test, (2) the C/B6a difference at four months to determine whether the average of the eight chips at four months is significantly non-zero, (3) the C/B6a difference at eight months, and (4) age dependency of the train differences to determine whether C/B6a at four months is significantly different from the C/B6a at eight months. For these four comparisons, p*-*values were tabulated and the false discovery rate (FDR) from Significance Analysis of Microarrays (SAM) was calculated. False discovery rate significance was set at < 0.05 for all genes.

### Gene ontology analysis

Using GenMAPP and MAPPFinder 2, Gene Ontology (GO) based overrepresentation analysis was performed on the differentially expressed genes, with FC≥1.2 or FC≤0.8 and FDR<0.05 [14]. Of the ~25,000 unique genes present on our microarrays, 9,318 were annotated with GO terms. Gene Ontology terms that were overrepresented in our list of differentially expressed genes were identified according to criteria published by Doniger et al. [15]. A ranked and filtered list of GO terms was generated using the following criteria: permutation p<0.01, Z score ≥2, percentage of genes changed >10%, and the number of genes changed ≥2. Regulated genes (FC≥1.2 or FC≤0.8 and FDR<0.05) are listed in Appendix 1.

### QTL analysis

The map position for each of the differentially expressed genes was determined [Mouse Genome Informatics] to identify genes that map to previously identified QTL associated with ageRD differences between these two strains [5].

### Candidate gene search using positional medline

Positional Medline (PosMed) is a database system created by the Riken Genomic Science Center (Yokohama, Japan) that creates a knowledge-based ranking of candidate genes within a chromosomal interval. The PosMed program searches for genes in a chromosomal interval that are connected to a keyword (specified by the user). According to PosMed, this connection is established by first identifying all the documents available in different document databases (MEDLINE abstracts, etc.) for the particular keyword. Then, the gene names or symbols mentioned in all the returned documents are extracted and a ranked list of candidate genes in the chromosomal interval is established based on a Fisher’s exact test. These additional PosMed candidate genes, which were identified independently of our microarray study, were subsequently cross-referenced on the NCBI website for eye-specific expression and the SAGE Retina Library for retina expression.

### Sequence analysis

Sequence analysis was performed bidirectionally using the Taq DiDeoxy Termination Cycle Sequencing kit (ABI, Foster City, CA). Eye-derived total RNA and cDNA was obtained as described above. Overlapping PCR fragments were obtained from the cDNA pool using Accuprime Pfx DNA polymerase (Invitrogen, Carlsbad, CA). The 5′ and 3′ ends of the mRNA were sequenced from PCR fragments obtained from genomic DNA. The PCR fragments were cloned in a pJET1.2/blunt vector (Fermentas, Glen Burnie, MD) before sequencing with pJET1.2 forward and PJET1.2 reverse primers (Fermentas). The observed SNPs were submitted to a dbSNP database.

The Mouse Genome Projects database and the GeneNetwork website were used to search for reported polymorphisms in genes inside the Chr 6 and Chr 10 QTL regions.

### Quantitative polymerase chain reaction validation of gene expression

Eyes were dissected as described above. Total RNA was obtained using RNA-bee (Tel-Test, Friendswood, TX). For each of the four groups (C and B6a, four and eight months of age), the eyes of three animals (six eyes) were pooled. The whole experiment from RNA extraction to real-time PCR was repeated three times with different pools of animals. The cDNA was obtained from 2 μg of total RNA primed with oligo dT using the Superscript III First-Strand Synthesis System kit (Invitrogen). The PCR amplifications were performed as described previously [16]. The PCR amplifications were conducted using the QuantiTect Syber Green PCR kit (Qiagen, Valencia, CA) with 0.01 U/μl of AmpErase UNG enzyme (Applied Biosystems, Foster City, CA) to prevent carryover contamination. Real-time PCR was performed in triplicate in a GeneAmp 5700 sequence detection system (Applied Biosystems) using the following cycling conditions: 50 °C for 2 min, 94 °C for 15 min, 40 cycles of 94 °C for 15 s, and 58 °C for 1 min. Quantitative values were obtained using the cycle number (Ct value), which is inversely proportional to the amount of a specific mRNA species in the tissue sample. Relative gene expression levels were calculated using the equation y=(1+AE)^ΔΔCt^, where AE is the amplification efficiency of the target gene (set at 1.0 for all calculations), and ΔΔCt is the difference between the mean experimental and control ΔCt values. The ΔCt value is the difference between the Ct value for a retina-associated gene and the *β-actin* internal reference control gene. The relative mRNA levels of B6a at four months were set at one (calibrator), and the levels of the other treatments were reported relative to it. The ANOVA test was performed to assess if the differences in the mean values of treatments are greater than would be expected by chance. If the ANOVA test was passed, pairwise multiple comparisons were performed using the Holm-Sidak method.

## Results

### Microarray analysis

Gene expression in posterior eyecups in the C and B6a strains at four and eight months of age was compared using DNA microarrays. The analysis of the transcriptional differences between the strains (especially at eight months of age when differences in ONL thickness are found) should reveal processes and pathways involved in ageRD.

Table 1 shows the GO terms overrepresented in each strain at four and eight months (regulated genes under each term are listed in Appendix 1). Interestingly, the GO terms overrepresented in C or B6a at eight months do not overlap, suggesting that different signaling and metabolic processes are taking place in the retinas of these two strains. In the C strain, GO terms associated with tissue remodeling (‘receptor-mediated endocytosis’, ‘laminin-1 complex’ and ‘cortical cytoskeleton organization and biogenesis’), stress and apoptosis (‘mitochondrial envelope’), mitosis (‘M phase of mitotic cell cycle’), signaling (‘GTPase activity’), and metabolism (‘carboxylic acid metabolic process’ and ‘porphyrin metabolic process’) were found (Table 1).

**Table 1:** GO term analysis of differentially expressed genes between the C and B6a strains at 4 and 8 months-of-age.

**GOID**	**GO Name**	**Number changed**	**Percent changed**	**Z score**	**Permute P statistic**
**GO terms increased in C versus B6a at 4 months**					
16628	oxidoreductase activity\, acting on the CH-CH group of donors\, NAD or NADP as acceptor	5	55.6	4.8	<0.001
30054	cell junction	25	21.9	4.746	<0.001
8305	integrin complex	7	41.2	4.6	<0.001
4716	receptor signaling protein tyrosine kinase activity	5	57.1	4.4	<0.001
44420	extracellular matrix part	15	21.7	3.6	<0.001
16769	transferase activity\, transferring nitrogenous groups	8	33.3	4.1	0.001
45111	intermediate filament cytoskeleton	11	26.8	3.9	0.001
6958	complement activation\, classical pathway	5	38.5	3.7	0.001
42060	wound healing	13	22.8	3.6	0.003
**GO terms increased in B6a versus C at 4 months**					
42401	biogenic amine biosynthetic process	6	35.3	4.4	<0.001
299	integral to membrane of membrane fraction	4	44.4	4.3	0.001
42398	amino acid derivative biosynthetic process	6	27.3	3.6	0.003
7222	Wnt receptor signaling pathway	4	40.0	4.0	0.004
46916	transition metal ion homeostasis	7	23.3	3.4	0.006
19886	antigen processing and presentation of exogenous peptide antigen via MHC class II	4	30.8	3.2	0.006
1664	G-protein-coupled receptor binding	8	20.51282	3.147	0.007
8009	chemokine activity	6	25.0	3.3	0.009
**GO terms increased in C versus B6a at 8 months**					
3924	GTPase activity	26	19.0	3.3	<0.001
6898	receptor-mediated endocytosis	8	32.0	3.6	0.001
5606	laminin-1 complex	4	57.1	4.1	0.002
5740	mitochondrial envelope	40	16.4	3.1	0.002
19752	carboxylic acid metabolic process	54	15.2	3.1	0.002
30865	cortical cytoskeleton organization and biogenesis	5	41.7	3.6	0.007
6778	porphyrin metabolic process	6	35.3	3.4	0.009
87	M phase of mitotic cell cycle	23	16.5	3.4	0.009
**GO terms increased in B6a versus C at 8 months**					
50880	regulation of blood vessel size	8	36.4	5.2	0
5720	nuclear heterochromatin	6	30.0	3.9	0.001
10038	response to metal ion	4	44.4	4.3	0.004
8083	Growth factor activity	12	14.0	3.1	0.004
19216	regulation of lipid metabolic process	5	26.3	3.2	0.007
1664	G-protein-coupled receptor binding	8	20.5	3.1	0.007
42773	ATP synthesis coupled electron transport	5	26.3	3.2	0.009

Gene ontology (GO) terms that are over-represented among differentially expressed genes meeting the criteria Fold of change>1.2 and FDR<0.05 for each comparison were determined using MAPPFinder 2. The list of GO terms reported below was generated using the following criteria: Permute p<0.01, Z score≥2, percentage of genes changed>10% and the number of genes changed at least 4 but lower than 100. Permute P is a non-parametric statistic based on 2000 permutations of the data.

In the ageRD-resistant B6a strain, GO terms related to vascular tone regulation (‘regulation of blood vessel size’), chromatin remodeling (‘nuclear heterochromatin’), stress (‘response to metal ion’), signaling (‘growth factor activity’, ‘G-coupled receptor binding’), and metabolism (‘regulation of lipid metabolic process’, ‘ATP synthesis coupled electron transport’) were observed (Table 1).

We interpret the results of the GO term analysis with care because the overrepresentation of a GO term in one strain indicates that mRNA expression levels of genes in that pathway or process are increased with respect to the other strain; but this does not necessarily mean that levels of protein expression are increased or that the pathway itself is activated because microarrays do not measure protein levels or post-translational modifications of proteins.

The upregulation of genes of the mitochondrial envelope in the C strain may reflect the increased apoptosis that occurs in this strain at eight months, as two pro-apoptotic genes (Bik and Bnip3l) are increased compared to B6a. The increase in cell remodeling is expected to be a consequence of apoptosis.

It is interesting to note that two antioxidant genes, Metallothionein 1 (*Mt1*) and Metallothionein 2 (*Mt2*; ‘response to metal ion’) are increased in the B6a strain, which could confer protection against ageRD.

**Figure 1:**
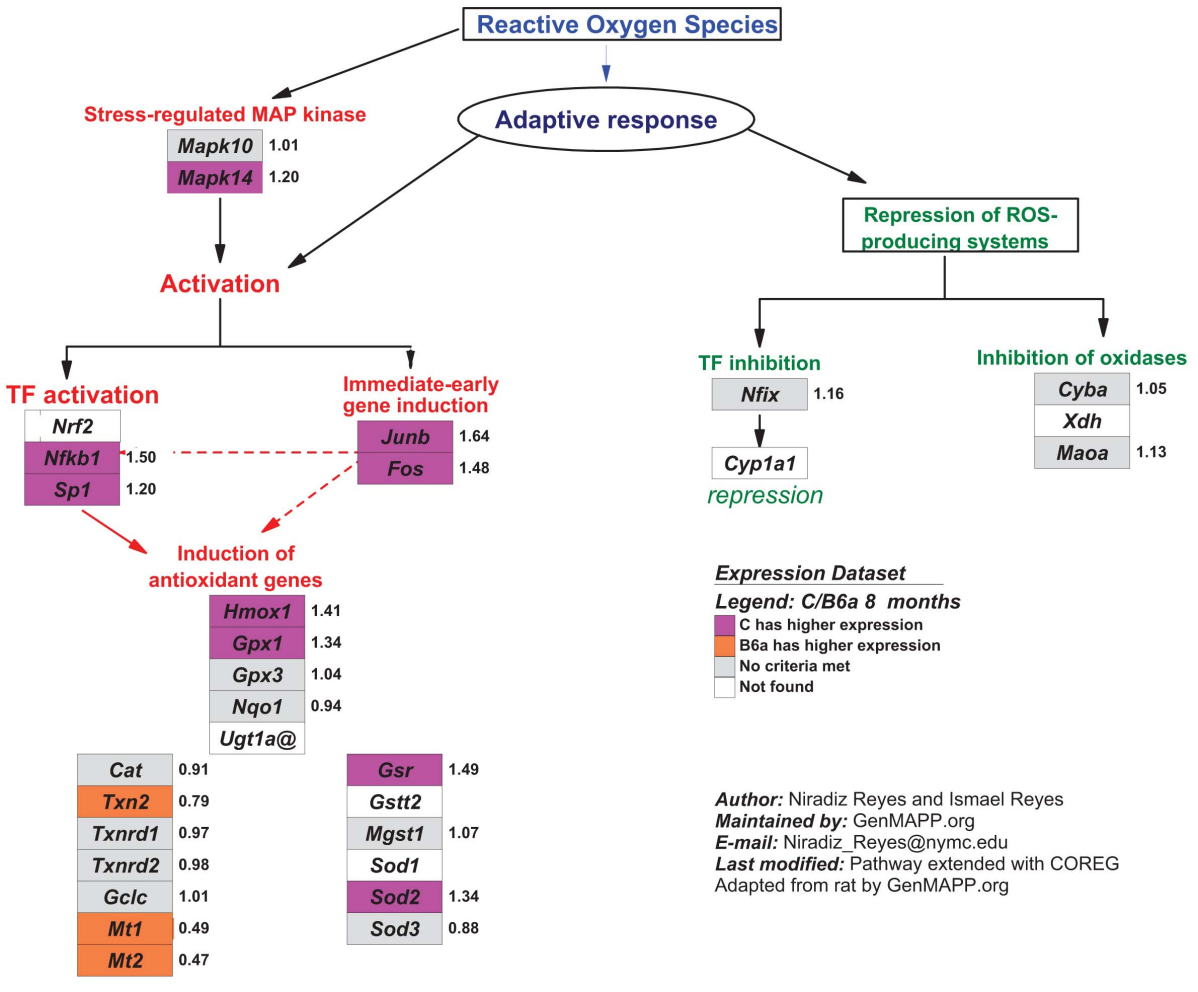
Oxidative stress MAPP pathway. The oxidative stress MAPP pathway was regulated at eight months (Z=2.366, permute p<0.01). Pink boxes indicate genes increased in C strain, FDR<0.05 and orange boxes indicate genes increased in B6a, FDR<0.05. Gray boxes indicate genes present in the data set, but not regulated. White boxes indicate genes not present in the data set. *Gsr*, *Mgst1*, *Cat*, *Mt1,* and *Mt2* were regulated only at four months. The pathway was created by Nirazid Reyes and Ismael Reyes and was adapted from Reference [39].

A pathway analysis (MAPPs) of the microarray was performed using GeneMAPP2. The pathways upregulated in C and B6a are in accord with the GO terms observed (data not shown). One of the pathways increased in C at eight months is ‘oxidative stress’ (Figure 1).

The hypothesis that both strains differ in the capacity to respond to stress could be postulated from the GO term analysis.

Finally, the differential expression of some of the genes was confirmed by qRT–PCR (Table 2).

**Table 2:** qRT–PCR confirmation of expression differences between strains for selected genes at 8 months.

**Name**	**GO term**	**Microarray**	**qRT–PCR**
*Hmox1*	Porphyrin metabolic process	1.4	1.2
*Sod2*	Mitochondrial envelope	1.3	1.2
*Mt1*	Response to metal ion	0.5	0.5
*Mt2*	Response to metal ion	0.5	0.6

Fold change values are expressed as C/B6a.

### Map positions of differentially expressed genes

In a previous quantitative genetics study about C and B6a, two highly significant QTL that influence ageRD were found in Chr 6 and Chr 10 [5]. The refined QTL on Chr 6 is localized to 77.5–93.0 Mb and to 15.4–20.8 Mb on Chr 10, encompassing 247 and 38 genes in these two intervals, respectively. Of these genes, 11 were found to be differentially expressed between the B6a and C strains for the Chr 6 QTL (Polymerase DNA directed epsilon 4 *(Pole4),* Chaperonin containing TCP1 subunit 7 *(Cct7),* RICKEN cDNA 1700019G17 gene *(1700019G17Rik),* Transforming growth factor alfa *(Tgfa),* Germ cell-less homolog 1 *(Gmcl1),* NFU1 iron-sulfur cluster scaffold homolog *(Nfu1),* Glutamine fructose-6-phosphate transaminase 1 *(Gfpt1),* Anthrax toxin receptor 1 *(Antxr1),* Gastrokine 1 *(Gkn1),* Minichromosome maintenance deficit 2 mitotin *(Mcm2),* Coiled-coil-helix-coiled-coil-helix domain containing 4 *(Chchd4)*), and four were found to be differentially expressed for the Chr 10 QTL (Coiled-coil domain containing 28A *(Ccdc28a),* PERP TP53 apoptosis effector *(Perp),* Peroxisomal biogenesis factor 7 *(Pex7), and* Myeloblastosis oncogene *(Myb)*; Table 3 and Table 4).

**Table 3:** Chr6 QTL candidate genes with SNPs fitting haplotype B≠C and A≠C.

**Gene**	**ID**	**Introns**	**Non-syn**	**Syn**	**UTR**	**PosMed**	**Microarray FC 4m**	**Microarray FC 8m**	**qRT–PCR FC 4m**	**qRT–PCR FC 8 m**	**N criteria**
*Fbln2*	Mm.249146	4				Yes	1.8	1			2
*Ctnna2*	Mm.34637	5				Yes	NP	NP			1
*Lrrtm4*	Mm.94135	27				No	1	1			0
*Tacr1*	Mm.8055	13				Yes	NP	NP			1
*Hk2*	Mm.255848	7				Yes	1	1	0.5*	1	1
*Sema4f*	Mm.270543	5				Yes	NP	NP			1
*Dok1*	Mm.156	1				Yes	1	1	1.2*	2.1*	1
*Htra2*	Mm.21880	2				Yes	1	1	1	1.3	1
*Vax2*	Mm.307165	1				Yes	NP	NP			1
*Cyp26b1*	Mm.255246	1	2			Yes	NP	NP			1
*Rab11fip5*	Mm.220334	1			No	1	1			0	
*Alms1*	Mm.246967	1			Yes	1	1			1	
*Tgfa*	Mm.137222	3				Yes	1.4	1.8			2
*Gmcl1*	Mm.321452	4				No	1	1.3			1
*Anxa4*	Mm.259702	32			1	No	1	1			0
*Aak1*	Mm.221038	133	1		3	Yes	1	1			1
*Nfu1*	Mm.23809	11	1		1	No	1	0.8			1
*Gfpt1*	Mm.19893	59	1			Yes	0.5	0.5			2
Antxr1**	Mm.232525	432		1		Yes	1.4	1	1	1	2
*Gkn1*	Mm.46414	2				No	1	0.9			1
*Arhgap25*	Mm.119564	85		1		No	NP	NP			0
*Ccdc48*	Mm.333229	1	1	1		NP	NP			0	
*Cnbp*	Mm.290251	1				1	1			0	
*Isy1*	Mm.241546	20			1	No	NP	NP			0
*Gata2*	Mm.476843			1		1	1			1	
*Eefsec*	Mm.333237	43				Yes	1	1	1	1	1
*Wnt7a*	Mm.56964	1				Yes	NP	NP			1
*Slc6a6*	Mm.395650	1				Yes	0.8	1			2
*Grip2*	Mm.333264	1				No	NP	NP			0

Number of criteria met for each gene (regulation found by microarray or presence in PosMed list) is indicated. FC ratios are C/B6a. NP represents not present in the microarray. **: Out of the 10 genes sequenced in exons, only the silent single nucleotide polymorphism (SNP) in Antxr1 fit the haplotype. *: Significant difference (p<0.05) in expression between strains obtained by qRT–PCR. Non-syn: nonsynonymous SNP. Syn represents synonymous SNP.

**Table 4:** Sequencing analysis of the selected Chr6 QTL candidate genes.

**Gene**	**Non-syn**	**Syn**	**5UTR**	**3UTR**
*Hk2*	0	T816C (ss161109849) G1130T (ss161109850)	0	(-)3885T (ss161109823)
* *		(-)1139TA (ss161109851) T1325C (ss161109852) T1573C (ss161109853) T1876C (ss161109854)		
*Dok1*	G45A (D2N) T301C (V87A)	T854C T1049C	C8G (ss161109825)	G1550(-) (ss161109830)
*Htra2*	C1381T (T449I) (ss161109831)	0	0	0
*Antxr1*	0	T472C** (ss161109833)	0	A2056G (ss161109834) C2057G (ss161109835)
*Eefsec*	0	0	0	0

NP represents not present in the microarray. **: Out of the 10 genes sequenced in exons, only the silent single nucleotide polymorphism (SNP) in Antxr1 fit the haplotype. Non-syn: nonsynonymous SNP. Syn represents synonymous SNP.

### Candidate gene search based on reported polymorphisms

To select candidate genes for further study, we looked for genes with reported polymorphisms between C and B6a in databases (The Mouse Genome Projects database and GeneNetwork website).

In a previous quantitative genetics study of age-related degeneration [6] between the strains B6 and A/J (A), several QTL were identified, but none matched with the QTL found in the cross between B6a (isogenic with B6) and C. Hence, genes that have SNPs of the haplotype B6≠C and C≠A would be very promising to investigate. In the Chr 6 QTL, we found SNPs in 59 genes and 29 genes with SNPs that fulfill the haplotype. In the Chr10 QTL, we found SNPs in 24 genes and 16 genes with SNPs that fulfill the haplotype (Table 3 and Table 5). In the Chr 6 QTL, SNPs fitting the haplotype were found either in the exons and introns of genes; in the Crh10, QTL SNPs fitting the haplotype were only found in the introns of genes.

**Table 5:** Chr10 QTL candidate genes with SNPs fitting the haplotype B≠C and A≠C.

**Gene**	**ID**	**Introns**	**PosMed**	**Microarray FC 4m**	**Microarray FC 8m**	**qRT–PCR FC 4m**	**qRT–PCR FC 8 m**	**N criteria**
*Heca*	Mm.473073	3	No	NP	NP			0
*AC153433.6*	ENSAMUSG95817	3	No	NP	NP			0
*Ccdc28a*	Mm.296565	19	No	1	1.3			1
*Nhsl1*	Mm.297971	1	No	1	1			0
*Hebp2*	Mm.35551	1	Yes	NP	NP	1.4	1.2	1
*Perp*	Mm.28209	2	Yes	2.8	1	1.2	1	2
*Tnfaip3*	Mm.116683	3	Yes	1	1	1	1.3*	1
*D10Bwg1379e*	Mm.425612	10	No	1	1			0
*Olig3*	Mm.156946	8	Yes	NP	NP			1
*Il20ra*	Mm.234667	6	No	NP	NP			0
*Pex7*	Mm.338363	1	Yes	0.8	1			2
*Map3k5*	Mm.6595	8	Yes	1	1	1.2	0.9	1
*Mtap7*	Mm.20928	8	Yes	1	1			1
*Bclaf1*	Mm.294783	3	No	1	1			0
*Pde7b*	Mm.425617	6	Yes	NP	NP			1
*Ahi1*	Mm.253280	5	Yes	NP	NP	1	1.1	1

NP represents not present in the microarray. *: Significant difference (p<0.05) in expression between strains obtained by qRT–PCR. Non-syn: nonsynonymous SNP. Syn represents synonymous SNP.

### Candidate gene search using Positional Medline

To supplement our search for gene candidates for mouse ageRD QTL on Chr 6 and Chr 10, we conducted a search for candidate genes using the PosMed database. Using the keyword “retinal degeneration,” a list of 24 genes was obtained for the Chr 6 QTL. When using the keyword “retin*,” an additional 41 genes were obtained (data not shown). The list of 24 genes contains two of the differentially expressed genes, *Gfpt1* and *Tgfa*. Using the keyword “retinal degeneration,” a list of seven genes was obtained for the Chr10 QTL. This increased to 12 genes when the keyword “retin*” was used. Only one gene in these two groups, *Perp*, corresponded to a differentially expressed gene in the microarray data.

### Sequence analysis and quantitative reverse transcriptase-PCR validation

We consider candidate genes as those that harbor SNPs and that fit the haplotype B6≠C and C≠A (Chr 6 QTL, 29 genes; Chr 10 QTL, 16 genes). We gave those candidate genes a score of two if they are present in both the PosMed list and are regulated in the microarray analysis, one if they are present in the PosMed list or are regulated in the microarray analysis, or zero if they do not fulfill either of the two criteria (Table 3 and Table 5).

Differences in the expression of genes and differences in protein activity between the two strains of mice might be due to the presence of polymorphic SNPs. To determine whether the presence of SNPs in the candidate genes accounts for the difference in susceptibility to ageRD in these strains, individual genes were sequenced. For practical purposes, sequencing was limited to five genes for each QTL. We selected genes with scores of two or one; the selection was also based on GO terms and the literature search.

In addition, qRT–PCR was performed to determine or verify differential expression of these genes between the C and B6a strains.

Sequence analysis and qRT–PCR results revealed polymorphisms and changes in expression levels for these candidate genes between the two strains (Table 3, Table 4, Table 5, and Table 6). Polymorphisms were identified in the coding regions of eight of the ten genes, and in the 5′ or 3′ UTRs of seven of the ten genes. The genes *Htra2,* Docking protein 1 *(Dok1)*, and *Tnfaip3* have nonsynonymous SNPs in exons. None of the nonsynonymous SNPs found fit the haplotype C≠B6, C≠A. By convention, we list the B6a amino acid first, followed by the position of the amino acid, followed by the C amino acid. One nonsynonymous SNP (I449T) was found in *Htra2*. The isoleucine allele found in B6a is conserved among other mouse strains. Two nonsynonymous SNPs were identified in *Dok1* (D2N and V87A), with the first B6a amino acid (D2) being conserved among other strains, and the second B6a amino acid (V87) not being conserved. Two nonsynonymous SNPs were found in *Tnfaip3* (E627A and L699P). Neither of the two B6a amino acids (E627 and L699) is conserved among other mouse strains. Only a synonymous SNP in Antxr1 fits the haplotype. Introns of the ten genes selected contained SNPs that fit the haplotype C≠B6, C≠A. Antxr1 and eukaryotic elongation factor selenocysteine-tRNA-specific (Eefsec) contain a large number of intronic SNPs.

**Table 6:** Sequencing analysis of the selected Chr10 QTL candidate genes.

**Gene**	**Non-syn**	**Syn**	**5UTR**	**3UTR**
*Hebp2*	0	C306T (ss161109845) C546G (ss161109846) G600A (ss161109847)	A22G (ss161109838) C44G (ss161109839) A124G (ss161109840) T147C (ss161109841) A198T (ss161109842) G208C (ss161109843) G219A (ss161109844)	G1687A (ss161109848)
*Perp*	0	0	0	T816C (ss161109849) G1130T (ss161109850) (-)1139TA (ss161109851) T1325C (ss161109852) T1573C (ss161109853) T1876C (ss161109854)
*Tnfaip3*	A2114C (E627A) (ss161109862) T2330C (L699P) (ss161109863)	0	G148C (ss161109861)	T4117G (ss161109864) T4123C (ss161109865) T4238C (ss161109866)
*Map3k5*	0	T437G (ss161109867) C926T (ss161109868) G3431A (ss161109869) C3483T (ss161109870)	0	0
*Ahi1*	0	G2346A (ss161109872)	0	(-)3323G (ss161109873)

NP represents not present in the microarray. **: Out of the 10 genes sequenced in exons, only the silent single nucleotide polymorphism (SNP) in Antxr1 fit the haplotype. Non-syn: nonsynonymous SNP. Syn represents synonymous SNP.

Changes in gene expression by qPCR were identified in *Hk2* and *Dok1* at four months and in *Dok1* and *Tnfaip3* at eight months. The remaining genes showed similar expression profiles between the two strains, as determined by qPCR.

### Outer nuclear layer phenotype of the Tnfaip3+/− mouse on a C57BL/6 background

*Tnfaip3* was selected for further study as a candidate gene for the Chr 10 QTL for ageRD based on the following observations. The *Tnfaip3* message of this anti-apoptotic gene was reported to be downregulated in different models of retinal degeneration [17]. *Tnfaip3* has been shown to be upregulated in B6 with respect to C using a different microarray platform (B. Rohrer, data not shown). We found no difference by microarray expression at either time point in either strain. However, using qRT–PCR, we found a 1.3 to 1.0 increase in C with respect to B6a at eight months, but no difference at four months (Table 5). We hypothesized that a higher activity of functional *Tnfaip3* protein in B6a confers protection against ageRD. Considering that a difference in ONL does not occur between the strains until between six and eight months, the upregulation of *Tnfaip3* in BALB/c may reflect overexpression of a misfunctional protein due to the two missense SNPs observed (Table 4).

**Figure 2:**
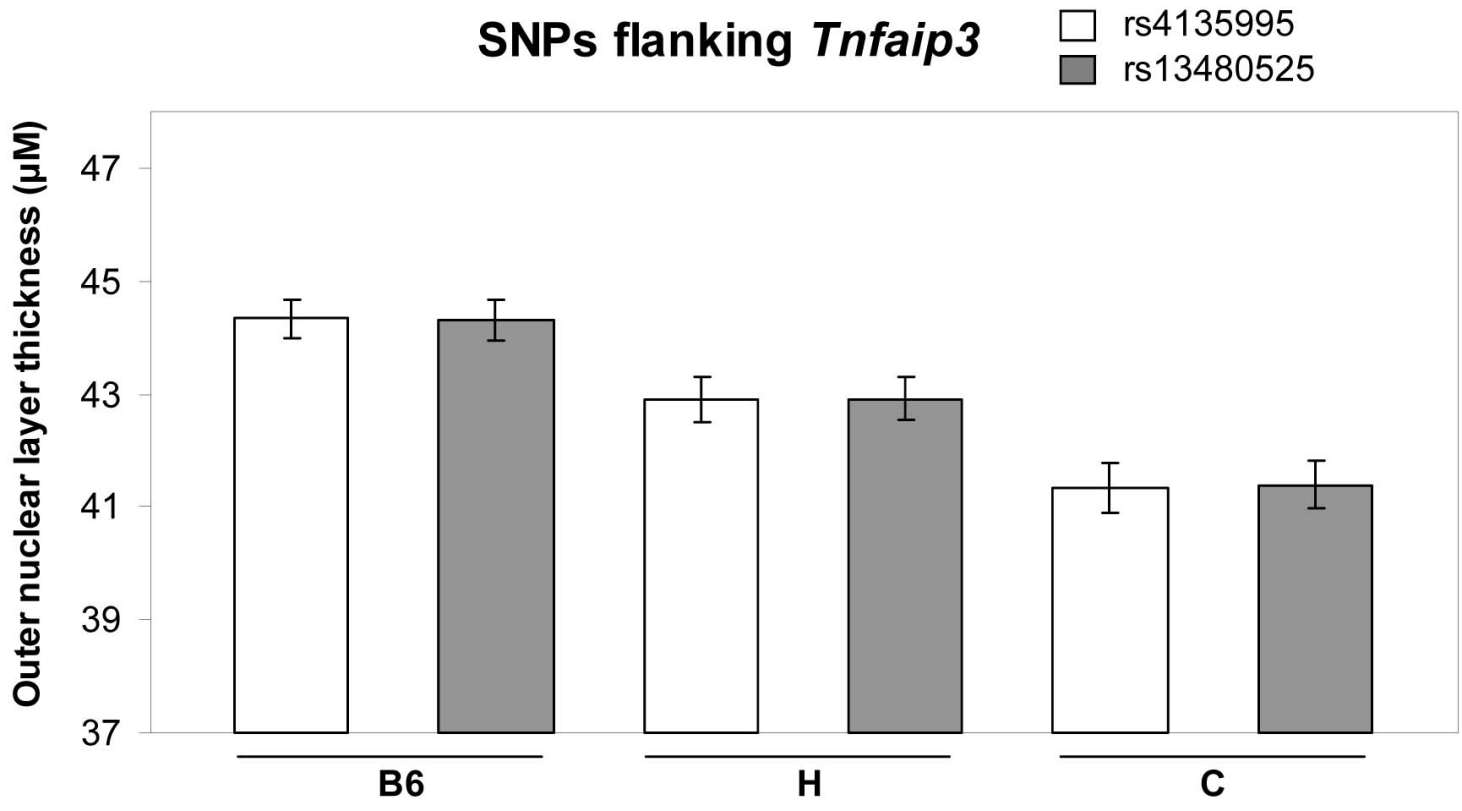
Analysis of SNPs flanking *Tnfaip3*. The outer nuclear layer thickness data were obtained from our previous study [5]. The phenotype of F2 animals (n=60), sorted by the genotype of two single nucleotide polymorphisms (SNPs) flanking *Tnfaip3*, is shown. The outer nuclear layer thickness of the B6, H (heterozygous), and C alleles for these two SNPs are significantly different (p<0.001). Mean and standard error of the mean are shown.

**Figure 3:**
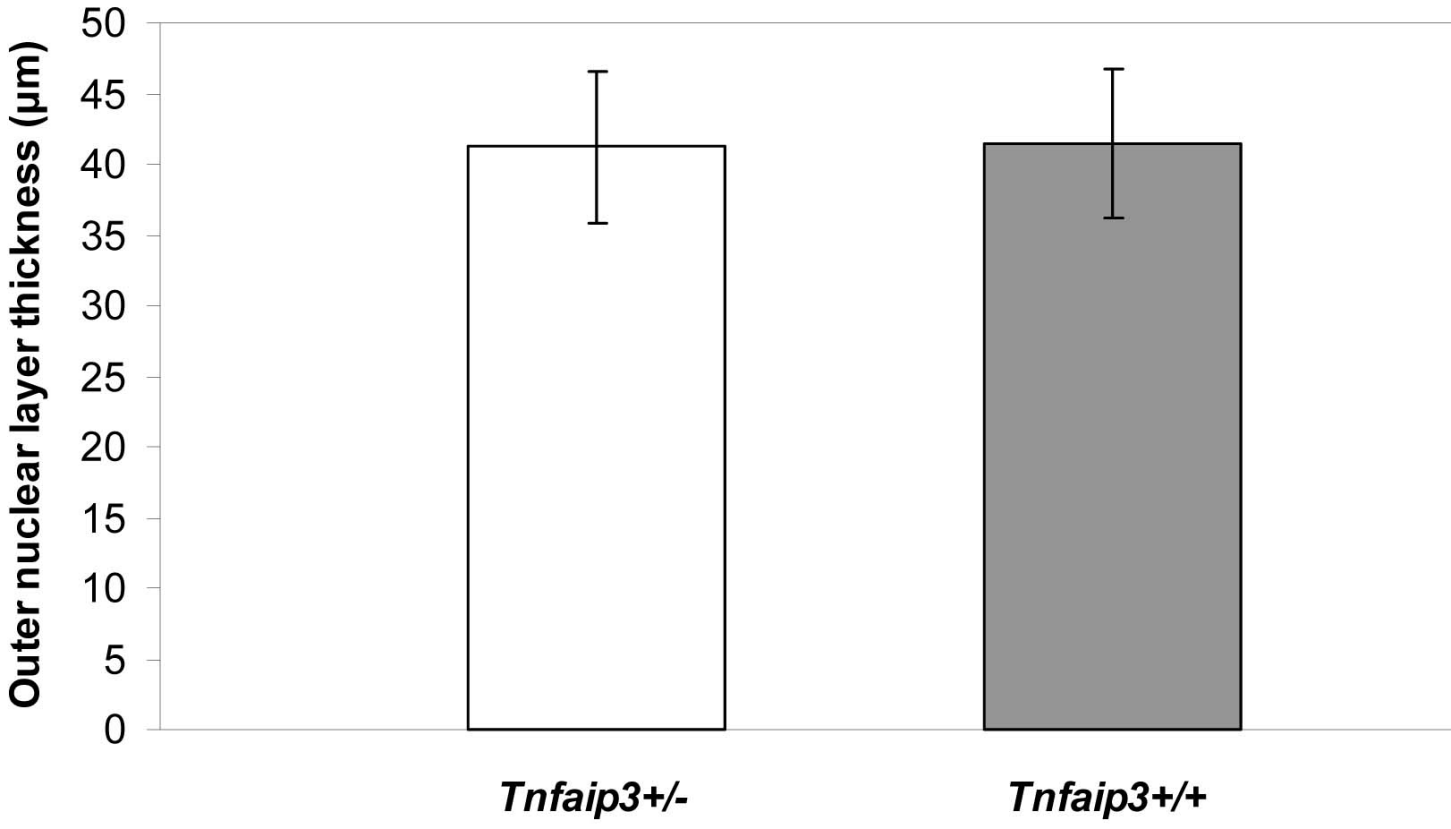
Phenotype of *Tnfaip3*^+/−^ mice on a B6 background (N8–10). Outer nuclear layer thickness was compared between *Tnfaip3*^+/−^ (n=12) and Tnfaip3^+/+^ mice (n=13).

SNP analysis was performed on the genomic regions flanking *Tnfaip3* on Chr 10, covering approximately 1.4 Mb downstream and 0.3 Mb upstream. The data were obtained from the quantitative trait analysis previously published [5]. The ONL thicknesses of eight-month-old F2 animals (n=60) carrying the B6a allele, the C allele, or both (heterozygous for B6a and C) were compared for the two SNPs that span *Tnfaip3*. The genotypes of the two SNPs flanking *Tnfaip3* (rs4135995 and rs1348525) are associated with significant differences in ONL thickness (Figure 2). Overall, an additive effect was observed, with the B6a allele being protective (Figure 2). We bred the *Tnfaip3* knockout allele onto the B6 background. The ONL thickness was compared in eight-month-old N8-N10 congenic *Tnfaip3^+/−^* and *Tnfaip3^+/+^* mice (*Tnfaip3*^−/−^ is lethal). However, no differences in ONL thickness could be identified (p=0.91) at eight months (Figure 3). Thus, the lack of an obvious retinal phenotype in *Tnfaip3*^+/−^ animals suggests that *Tnfaip3* is not the QTG for the Chr 10 QTL.

## Discussion

Using a combination of gene expression, bioinformatics, and sequencing approaches, we were able to identify candidate genes for the Chr 6 and Chr 10 QTL responsible for the difference in the ageRD phenotype between the C and B6a mouse strains. The GO term analysis of the competitive microarray revealed pathways and processes associated with ageRD.

### Identification of candidate genes

The haplotype analysis identified 35 candidate genes for the Chr 6 QTL and 14 for the Chr 10 QTL. Those genes were scored whether they were present in PosMed or regulated by microarray analysis. For the sake of practicality, we selected only five candidate genes per QTL with scores of 2 or 1 for further analysis, based on the literature search. Based on our previous study [6], the QTG alleles need to fit the haplotype C≠B6, C≠A. Even though this haplotype is very likely, it is also possible that polymorphisms that do not fit the haplotype (C≠B6, C=A) produce a phenotype in the C background and not in the A background. Hence, it is possible that our analysis ruled out some possible candidate genes. Conversely, our sequencing results identified SNPs that do not fit the haplotype, but for the reason mentioned above were not ruled out as being responsible for the phenotype.

Sequence analyses revealed a wide spectrum of polymorphisms between the B6a and C strains in the coding region as well as in the 5′UTR and 3′UTR and 5′ genomic and 3′ genomic DNA regions (Table 3 and Table 6). A qRT–PCR revealed differences in gene expression between the B6a and C strains for *Hk2, Dok1*, and *Tnfaip3* (Table 3 and Table 5). The remaining seven genes showed similar mRNA expression profiles between the two strains. Protein expression differences may be expected due to the presence of SNPs in the UTRs. Our results enabled us to postulate testable hypotheses for three of the ten genes: *Htra2, Dok1, and Tnfaip3.*

High temperature requirement protein A2 (*Htra2*) encodes a serine protease. No differences in gene expression between the strains were identified. Differences in protein activity may be caused by a nonsynonymous SNP; the B6a amino acid is conserved among other mouse strains (but do not fit the haplotype). The *Htra2* protein has been shown to play a role in mitochondrial homeostasis [18]. Polymorphisms in *Htra2* are associated with Parkinson disease and neuronal degeneration [19]; the *Htra2* knockout mouse has severe neuronal degeneration [18]. The hypothesis is that the C allele is associated with a lower capacity of the mitochondria to cope with stress. Studies of *Htra2* knockout mice will be performed.

*Dok1* acts downstream of a receptor tyrosine kinase. *Dok1* expression is decreased in B6a with respect to C at eight months. Differences in protein activity could be caused by either or both of two nonsynonymous SNPs found in the coding region (Table 4). The first B6a amino acid (D2) is conserved among species, but the second B6 amino acid (V87) is not. Any of both fit the haplotype B6≠C and C≠A mentioned above. *Dok1* protein is associated with activation, proliferation, and migration in the nervous system [20] and the results from the *Dok1*/*Dok2* double knockout mice suggest that the two proteins promote apoptosis [21]. The hypothesis is that a lower expression or activity of *Dok1* in B6a protects against apoptosis. More studies of *Dok1* knockout mice will be performed in the future.

*Tnfaip3* (tumor necrosis factor, alpha-induced protein 3) is a zinc finger protein, the expression of which is rapidly induced in response to tumor necrosis factor [22]. *Tnfaip3* mRNA expression was found to be increased in both strains at eight months compared to four months. At eight months, the expression in C is 1.3 to 1.0 compared to that of B6a, as revealed by qRT–PCR. No differences between the two strains were found by DNA microarray analysis at either time point. *Tnfaip3* has been shown to be upregulated in B6 with respect to C using a different microarray platform (B. Rohrer, unpublished results). However, qRT–PCR results are generally thought to be more reliable than DNA microarray results. This anti-apoptotic gene has been shown to be downregulated in different models of retinal degeneration, such as bright light exposure and elimination of endothelin-2 [17]. Differences in protein activity may be caused by one or both of the two nonsynonymous coding SNPs (Table 5). Neither amino acid (E627 or L699) is conserved among strains, nor do they fit the haplotype. The hypothesis is that a higher activity of *Tnfaip3* in B6a confers protection against retinal degeneration.

Based on the gene expression data suggesting the involvement of *Tnfaip3* in both genetic and environmental models of photoreceptor degeneration, we decided to study *Tnfaip3* as a candidate QTG for the Chr 10 locus further. Two SNPs flanking *Tnfaip3* (rs4135995 and rs13480525) were correlated with ONL thickness. The ONL thickness was positively correlated with the B6a allele, with intermediate cell survival in the heterozygote animals, demonstrating an additive behavior (Figure 2). However, when photoreceptor survival was analyzed in eight-month-old *Tnfaip3*^+/−^ animals, no difference in ONL thickness could be identified in N8-N10 congenic heterozygous and wildtype mice (Figure 3). This lack of phenotype in *Tnfaip3*^+/−^ animals suggests that *Tnfaip3* is not the QTG for the Chr 10 QTL. However, the correlation of Tnfaip3 SNPs and ONL differences may indicate that the actual QTG is close to this gene.

Of the ten genes selected for study, two more genes were ruled out as candidate genes: *Ahi1* and *Antxr1*. *Ahi1* is involved in retinal degeneration in human subjects [23]. We found that it does not harbor non-synonymous SNPs, but contains SNPS in introns that fit the haplotype. Using qRT–PCR, we found no differences that might rule out this gene as a candidate (Table 5). Another gene that harbors a great number of SNPs in introns fitting the haplotype is Antxr1. We did not find differences in expression using qRT–PCR (Table 5). For this gene, we detected one splicing isoform that was expressed at the same level in both strains (not found) using northern blotting. It was then ruled out as a candidate.

In future studies, genes that harbor non-synonymous mutations that fit the haplotype, such as Cyp26b1 and Gfpt1, need to be considered.

### Pathway analysis in age-related degeneration

Gene Ontology analysis of the differentially expressed genes in ageRD was performed to identify possible processes and pathways involved.

In the ageRD sensitive C strain, genes of the mitochondrial envelope were upregulated and were probably directly related with the apoptosis mechanisms present in this strain at eight months. Two of those genes, *Bik* and *Bnip3l*, are pro-apoptotic [24,25]. Gene Ontology terms associated with cell remodeling are increased in C. Neural remodeling is a typical consequence of photoreceptor death [26].

Even though oxidative stress-related GO terms themselves do not appear in the ranked list of GO terms for C at eight months, the MAPP pathway ‘oxidative stress’ was overrepresented in this strain at eight months (Figure 1). Genes involved in protection against oxidative stress [27-30] are found inside this pathway: *Sod2, Hmox1, Gss,* and *Gpx1* (Figure 1). This may indicate that the retina of the C strain at eight months of age has an increased sensitivity to oxidative stress compared to the B6a strain. This hypothesis is supported by experiments using the paraquat-induced generation of free radicals, which revealed greater sensitivity of the C-strain retinas when compared to those from the B6 strain [31]. There is a strong link between oxidative stress and aging in the retina [32].

In the ageRD resistant B6a strain, the GO term ‘response to metal ion’ is overrepresented at eight months. The genes *Mt1* and *Mt2* inside this term have increased expression in B6a relative to C (Appendix 1, associated with the GO term “response to metal ion”). *Mt1* and *Mt2* are powerful antioxidants [33-37], suggesting that the B6a retina has elevated basal levels of antioxidants, making the photoreceptors more resistant to permanent exposure to reactive oxygen species generated by the absorption of light.

Genes involved in the regulation of vascular tone are induced in B6a relative to C (Appendix 1, ‘regulation of vasoconstriction’). Since only the blood vessels in the retina and not those of the choroid can autoregulate [38], changes in gene expression should reflect an adjustment of blood flow for the oxygen requirement of the retina. However, as two vasoconstrictor genes (*Agt*, *End1*) and two vasodilator genes (*Calca*, *Atp1a2*) had increased expression in B6a relative to C, the net effect on vascular tone could not be predicted with these results.

The remaining GO terms increased in both strains, pointing to differences in metabolism and signaling.

We interpret the results of the GO term analysis with care because the overrepresentation of a GO term in one strain indicates that mRNA expression levels of genes in that pathway or process are increased with respect to the other strain. However, this does not necessarily mean that levels of protein expression are increased or that the pathway itself is activated because microarrays do not measure protein levels or post-translational modifications of proteins.

### Conclusions

In summary, we have discussed a functional genomics approach, combining SNP haplotype analysis, gene expression using microarrays, and database mining to identify novel candidate genes for QTL associated with ageRD. SNP analysis proved to be a useful tool to refine the search for promising candidate genes, although in the future studies of polymorphisms in transcription regulatory regions need to be performed.

Further studies are now needed to provide more evidence of the functionality, role, and relevance of these genes. These studies include sequencing of the genes in the two strains and the generation of appropriate knockout mouse strains, or elimination/activation of the targeted gene or pathway by pharmacological or molecular means. We hope to test these and other hypotheses in the future.

One of the main hypotheses that could be extracted from the GO term analysis is that the two strains have a different capacity to cope with oxidative stress.

Finally, the identification of modifier genes/alleles that influence the course of ageRD in mice will provide human gene candidates that may influence the course of monogenetic RD phenotypes and complex genetic phenotypes like AMD.

## Appendix 1. Gene Ontology based overrepresentation analysis of microarray.

To access the data, click or select the words “Appendix 1.” This will initiate the download of a compressed (pdf) archive that contains the file.
